# The effects of digital economy development on social insurance funds revenue: Evidence from China

**DOI:** 10.1371/journal.pone.0303897

**Published:** 2024-05-21

**Authors:** Xiaoqing Pan, Bo Li, Jing Wu

**Affiliations:** 1 School of Political Science and Law, Zhengzhou University of Light Industry, Zhengzhou, Henan, China; 2 School of Public Administration, Zhongnan University of Economics and Law, Wuhan, Hubei, China; 3 School of Public Administration, Yanshan University, Qinhuangdao, Hebei, China; Sheikh Hasina University of Science and Technology, BANGLADESH

## Abstract

China has experienced rapid development in the digital economy. Using data from 30 provinces in China between 2011 and 2017, this paper constructs a two-way fixed effects model to study the effects and mechanisms of the digital economy development on social insurance funds revenue. An increase of one unit in digital economy development led to a 0.56% increase in basic endowment insurance funds revenue and a 0.33% increase in basic health insurance funds revenue. The digital economy increased the social insurance funds revenue by promoting employment and increasing income. Furthermore, the effects of digital economic development on social insurance funds revenue were heterogeneous for different levels of economic development and urbanization. The conclusions stood after robustness tests by changing the method of weighting the digital economy indicators and using instrumental variables. This paper confirmed the positive role of the development of the digital economy in increasing the revenue of social insurance funds from the perspective of quantitative research and explored the mechanisms in depth. In order to increase social insurance funds revenue, it is essential to accelerate the development of the digital economy, especially in regions with lower economic development and urbanization, and to address the needs of the technically unemployed and those engaged in flexible employment.

## 1. Introduction

Adequate social insurance funds are crucial for the sustainable development of social insurance. Population aging has caused financial challenges for social security systems in developed countries. China, as one of the fastest aging developing countries [1, 2], also faces the risk of insufficient social insurance funds revenue to cover expenditure [3]. Since 2014, China’s basic endowment insurance expenditure has exceeded the contribution revenue, leading to a reliance on fiscal subsidies to maintain funds balance. The growing proportion of elderly people further increases the risk of overspending on the basic health insurance funds. Therefore, it is essential to explore ways to increase revenue in order to maintain funds balance amidst rigid expenditure growth.

The digital economy is a new economic model that integrates and expands traditional sectors by using digital information and digital technologies, such as big data, artificial intelligence, and cloud computing, as key production factors [4, 5]. China has become a leading player in the international digital economy field, with the size of digital economy reaching 50.2 trillion yuan in 2022, ranking second in the world. The digital economy serves as a core driver of China’s economic development and has had a positive impact on green innovation, manufacturing productivity, agriculture, and sustainable supply chain performance [6–9]. However, the potential positive impact of the digital economy on social insurance funds revenue has not been extensively explored through quantitative research.

Social insurance funds revenue depends on factors such as the participation rates and contributions per capita, which are influenced by employment and income. The digital economy has a dual effect on employment, as it can lead to technological unemployment while at the same time creating new jobs [10]. Previous research has not reached a consensus on whether the digital economy increases net employment [11, 12]. In addition, while the digital economy creates formal jobs, it also creates flexible jobs that are not covered by the social insurance system [13]. In China, although flexible workers can contribute to social insurance, their unstable income and the lack of mandatory incentives to participate may limit their contributions [14]. In terms of income, the digital economy may increase income and contribution capacity by promoting labor mobility and skill upgrading [15, 16]. However, the unpredictable income of flexible workers may discourage social insurance contributions [17].

In summary, it is challenging to determine theoretically whether the digital economy has a positive impact on social insurance funds revenue and, if so, how the employment and income mechanisms work. This paper has taken basic endowment insurance and basic health insurance as research objects and empirically examined the impact of the development of the digital economy on social insurance funds revenue.

There are three highlights in this paper. First, it identifies the new factor affecting the social insurance funds revenue from the perspective of technological change, and deepens the understanding of the changing trend of the social insurance funds revenue in the digital era. Second, it analyzes the employment and income mechanism of the digital economy affecting the revenue of the social insurance funds from both theoretical and empirical perspectives. Third, it provides policy insights on how to improve social security revenue in the digital economy era.

## 2. Literature review

### 2.1 Study on the impact of the digital economy

Driven by digital technology, the digital economy has become a new engine for promoting economic and social development, with far-reaching effects on green development, innovation development, and income distribution. In terms of green development, Zhao et al. (2023) based on the data from prefecture-level cities in China, found that the development of the digital economy is conducive to improving the green total factor productivity (GTFP) of cities [18]. The upgrading of enterprises’ production technology and the exit of polluting enterprises are important mechanisms through which the digital economy affects GTFP. Based on the spatial Durbin model test, Wang et al. (2023) found that the digital economy significantly promotes the improvement of carbon emission performance and has a spatial spillover effect [19]. Xu et al. (2022) investigated the spatial interactive spillover effect of the digital economy and environmental pollution, and found that the digital economy and environmental pollution inhibit each other and have significant spatial spillover effects [20]. In terms of innovation development, Xu and Li (2023) found that the digital economy has a facilitating effect on innovation output, improving the quality of innovation output more than the quantity of innovation output [21]. The digital economy facilitates the digital transformation of enterprises and thus has a facilitating effect on enterprise innovation [22]. In terms of income distribution, the digital economy plays an important role in promoting common wealth, and the lower the level of the digital economy, the greater the promotion of common wealth. There is a U-shaped relationship between the digital economy and the urban-rural income gap [23]. Although the digital economy has boosted the income of China’s rural residents, it has widened the income gap within rural areas [24]. The digital economy has also played an important role in raising the incomes of rural migrants [25].

### 2.2 Study on the factors affecting the revenue of the social insurance funds

Maintaining the balance of the social insurance funds is of great importance. Exploring the factors that affect the income of social security funds and how social insurance systems can be reformed in order to achieve sustainability are important concerns in the existing literature. The defined contribution pension insurance system is vulnerable to shocks from demographic and economic fluctuations. Devolder et al. (2020) proposed an automatic equalization mechanism to address the financial sustainability of pension insurance. The source of revenue for the social insurance system depends on payroll taxes, which essentially depend on the employment rate and wage levels [26]. Despite the significant reform of the health insurance system in Croatia since 2002, the level of financial protection provided by the system remains low due to the lack of expansion of the fund’s revenue sources and improvement of the efficiency of tax collection [27]. Population aging may threaten the balance of pension and health insurance funds [28, 29]. In recent years, the growth rate of pension funds balance in China has been decreasing year by year and there are large regional differences in the funds balance, and factors such as the number of working age population and the population aged 65 years and above are the main factors affecting the regional differences in the pension funds balance [30]. Nishimura (2022) examines the optimal levels of public pensions and public long-term care (LTC) insurance, and it is always desirable to balance the budget to increase LTC benefits at the expense of public pension benefits [31]. Economic growth and political stability not only affect the stability of the state, but also put the pension fund balances in a critical state [32].

## 3. Materials and methods

### 3.1 Study setting and design

After the 1990s, China established a mandatory social insurance system for urban workers, including basic endowment insurance, basic health insurance, maternity insurance, unemployment insurance, and work injury insurance. Specifically, the basic endowment insurance guarantees the basic livelihood of the insured after retirement, while the basic health insurance distributes the health risks of employees. The basic endowment insurance and basic health insurance are the two most important components of the social insurance system due to their broadest coverage and highest rates. Therefore, this paper selected the revenue of the basic endowment insurance funds and the revenue of basic health insurance funds as two indicators to analyze the revenue of China’s social insurance funds.

#### Two-way fixed effects model

This article is a quantitative study of whether the development of the digital economy is the cause of the growth of the social insurance funds revenue, based on the method of regression analysis. We collected panel data covering 30 provinces in China from 2011 to 2017. The pooled model, fixed effects model, and random effects model are models that can be applied to panel data. The fixed effects model was found to be appropriate for this research based on the results of the F-test and Hausman test. The two-way fixed effects model is a powerful tool for analyzing panel datasets because it can address the endogeneity problem caused by omitting factors that do not vary across individuals and over time by controlling for individual fixed effects and year fixed effects, and it can also improve robustness to unobserved heterogeneity [33, 34]. We included both individual-fixed effects and time-fixed effects to address endogeneity due to unobservable provincial characteristics and time trends. The two-way fixed effects model was therefore chosen. Eqs (1) and (2) show the model:

$${EIFR}_{it}=\alpha_{0}+\alpha_{1}{Dige}_{it}+\alpha_{c}Z_{it}+\mu_{i}+\delta_{t}+\varepsilon_{it}$$ (1)

$${HIFR}_{it}=\alpha_{0}+\alpha_{1}{Dige}_{it}+\alpha_{c}Z_{it}+\mu_{i}+\delta_{t}+\varepsilon_{it}$$ (2)

In Eqs (1) and (2), *i* and *t* stand for province and time, respectively. *EIFR* and *HIFR* are explained variables denoting the basic endowment insurance funds revenue and the basic health insurance funds revenue, respectively. *Dige* is the core explanatory variable. *Z* denotes a set of control variables. The year-fixed effects are indicated by δ, while the individual (province)-fixed effects are denoted by μ. *α*_*0*_, *α*_*1*_ and *α*_*2*_ are the parameters that need to be estimated. The unobservable random disturbance term is represented by *ɛ*.

### 3.2 Variables design

#### 3.2.1 Explained variables: Basic endowment insurance funds revenue (*EIFR*) and basic health insurance funds revenue (*HIFR*)

The total amount that local governments collect annually for basic endowment insurance and basic health insurance is known as the basic endowment insurance funds revenue and basic health insurance funds revenue. This data is publicly available, and we can obtain it from the official website of the National Bureau of Statistics of China.

#### 3.2.2 Core explanatory variable: Digital economy (*Dige*)

Most scholars measure the digital economy from multiple perspectives using composite indicators. In this paper, based on Zhao et al. [33], five secondary indicators were selected from the two dimensions of Internet development and digital transaction development to construct the digital economy development index system (see Table 1). The instructions for calculating each indicator are given in the last column of Table 1. The data used include Internet users, computer services and software workers, workers in urban units, the total number of telecommunication services, mobile phone users, population, and the digital inclusion financial index. These data are all available through open sources. For information on how the data were collected, see the section on data collection and analysis.

**Table 1:** Digital economy development evaluation index system.

First-level indicators	Second-level indicators	Calculation instructions
Internet development	Internet penetration rate	Internet users per 100 people
	Related practitioner profile	Computer services and software workers accounting for the proportion of the employed in urban units
	Related outputs	Total telecom services per capita
	Cell phone penetration rate	Number of mobile phone subscribers per 100 people
Digital transaction development	Digital inclusive finance	digital inclusive finance index

The second-level indicators were measured according to the data obtained and the calculation instructions. The weight of each indicator in this research was determined using the entropy weight method. The entropy weight method is an objective method that determines the weight of indicators through the magnitude of the indicator’s variability. The digital economy development index, which represents the level of development of the digital economy, was calculated by adding the weighted values of these indicators.

#### 3.2.3 Control variables

Besides the digital economy, there exist other factors that could affect the social insurance funds revenue. Referring to the standard procedure in the current literature [19], the following variables were added as control variables in order to reduce the bias of the estimation results caused by the omitted variables:

*Industrial structure* (*Ind*). Industrial structure refers to the composition of industries in a country or region in the process of social reproduction. The relative proportions of industries vary at different stages of social production. A more advanced industrial structure implies potentially higher income levels and greater capacity to contribute to economic growth. In this paper, industrial structure has been expressed in terms of the ratio of value added in the tertiary sector to that in the secondary sector.

*Degree of openness to the outside world* (*Doow*). This variable reflects how open the economy of a country or region is to the outside world. The measure of openness to the outside world was expressed as the GDP divided by the total amount of imports and exports. The volume of imports and exports can affect employment and wages [35, 36], which in turn can affect the amount paid into social insurance.

*Fiscal expenditure* (*Fin*). Absolute fiscal expenditure is the amount of fiscal resources actually allocated and used by the government during the fiscal year. Relative fiscal expenditure is the amount of absolute fiscal expenditure relative to a given variable. In order to make the variable comparable across samples, relative fiscal expenditure is used in this paper. We measure the fiscal expenditure by dividing the fiscal general public budget expenditure by regional GDP. Fiscal expenditure can increase the availability of public goods and attract new workers [35–37]. Fiscal expenditures related to social security and employment contribute to employment growth and wage increases, which affect the social insurance funds revenue.

*Urbanization* (*Up*). Urbanization refers to the process by which rural populations become urbanized. We used the urbanization rate to represent the degree of urbanization. The proportion of the total population living permanently in urban areas can be used to define the urbanization rate. An area’s degree of urbanization correlates with the number of workers in the non-farm sector, which affects social insurance contributions and funds revenue.

*The aging rate* (*Ar*). Population ageing is a dynamic process in which the proportion of older people in the total population increases. The aging rate referred to the proportion of the population aged 65 years and above to the total population. A larger elderly population means fewer people working and contributing to social insurance.

#### 3.2.4 Mediating variables

The revenue of social insurance funds is determined by the number of contributors and the amount of contributions per capita. With the development of the digital economy, employment opportunities are increasing and wages are rising, resulting in a higher number of potential contributors and a greater capacity to contribute. Therefore, this study aims to examine the impact of the digital economy on social insurance funds revenue through employment and income, with the employment rate and average wage serving as mediating variables.

*Employment rate (Emp)*. The employment rate is an indicator of the extent to which the labor force is employed. We measured the employment rate by calculating the ratio of the number of employed individuals to the population aged 15 and over. An increase in the employment rate indicates that the digital economy can create more job opportunities and reduce unemployment. As a result, the overall increase in employment would lead to a larger pool of contributors.

*Average wage (Aw)*. The average wage is the average amount of wages and salaries received by each employee over a period of time. Income serves as the economic basis for participation in social insurance, and higher income leads to higher social insurance contributions within the maximum contribution limit. The digital economy affects wages by influencing the structure of supply and demand in the labor market. Compared to the non-private sector, the private sector is more sensitive to market forces. Consequently, the digital economy is more likely to affect the private sector wages, which in turn affect the contribution behavior of private sector employees. Therefore, we focused on the average wage of employees in the private sector.

### 3.3 Data collection and analysis

In this study, we manually collected data from publicly available data sources. With the exception of the digital inclusive finance index, all other variables were obtained from the China Statistical Yearbook. The China Statistical Yearbook is an annual publication compiled and published by the National Bureau of Statistics of China. It provides comprehensive statistics on various aspects of the economy and society for the whole country, as well as for provinces, autonomous regions, and municipalities directly under the central government. Data are collected through sample surveys or censuses conducted by survey teams from the National Bureau of Statistics of China and local statistical organizations, and are available on the official website of the National Bureau of Statistics of China. The digital inclusive finance index used in this study was taken from the report "Peking University Digital Inclusive Finance Index", published by the Digital Finance Research Center of Peking University.

This study collected panel data from 30 provinces (municipalities and autonomous regions) in China for the period of 2011–2017, resulting in a total of 210 samples, as detailed in the supporting information (S1 Data). However, the data for Tibet, Hong Kong, Macau, and Taiwan were not included due to severe missing data in these regions.

The collected data were analyzed using Stata software, version 15. First, the collected data were imported into the Statas software. Second, the F-test and Hausman test commands were used to determine the appropriateness of the two-way fixed effects model for this study. Third, estimates of *α*_*0*_, *α*_*1*_, and *α*_*2*_ were obtained by inputting Eqs (1) and (2) into the Stata software using commands.

The main focus of our analysis was the estimated coefficient *α*_*1*_ of the core explanatory variable *Dige*. If *α*_*1*_ was found to be positive and significant, at least at the 10% statistical level, we could conclude that the digital economy made a significant positive contribution to the revenue of social insurance funds.

## 4. Empirical results

### 4.1 Statistical characteristics of the sample

We collected 210 samples and identified ten variables. Specifically, the values of the variables *EIFR*, *HIFR*, and *Aw* were logarithmized to solve the problem of heteroskedasticity. The descriptive statistical characteristics of these variables, including the number of observations (obs.), mean, standard deviation (std. dev), and minimum (min) and maximum (max) values, are shown in Table 2.

**Table 2:** Descriptive statistics of variables.

variables		obs.	mean	std. dev	min	max
**Explained variable**	*EIFR*	210	6.514	0.839	4.274	8.148
	*HIFR*	210	5.270	0.854	3.000	7.201
**Explanatory variable**	*Dige*	210	0.644	0.344	0.056	1.990
**Control variable**	*Ind*	210	1.085	0.617	0.518	4.237
	*Doow*	210	0.255	0.292	0.016	1.440
	*Fin*	210	0.245	0.102	0.110	0.627
	*Up*	210	0.566	0.124	0.350	0.896
	*Ar*	210	0.098	0.020	0.055	0.143
**Mediating variable**	*Emp*	210	0.699	0.078	0.510	0.892
	*Aw*	210	10.388	0.266	9.725	11.167

### 4.2 Benchmark regression

Based on the two-way fixed effects model (as shown in Eqs (1) and (2)), regressions were run using Stata software to obtain the benchmark regression results, as shown in Table 3. Columns (1) and (3) of Table 3 show the effect on the basic endowment insurance funds revenue, while columns (2) and (4) present the effect on the basic health insurance funds revenue. No control variables are added in columns (1) and (2) and all control variables are added in columns (3) and (4). The R-squared is a measure of goodness of fit and indicates the extent to which the explanatory variables, control variables, and fixed effects (including individual-fixed effects and year-fixed effects) in the model explain the explained variables. In all four columns, the R-squared is greater than 0.9, indicating that the model explains the data well.

**Table 3:** Benchmark regression results.

Variables	(1)	(2)	(3)	(4)
	*EIFR*	*HIFR*	*EIFR*	*HIFR*
***Dige***	0.434**	0.311***	0.555***	0.332***
	(2.261)	(2.948)	(2.840)	(3.108)
***Ind***			−0.166**	−0.166***
			(−2.207)	(−4.027)
***Doow***			0.058	0.047
			(0.610)	(0.902)
***Fin***			−0.384	−0.181
			(−0.613)	(−0.528)
***Up***			−1.149	−0.642
			(−1.347)	(−1.377)
***Ar***			−4.278***	−0.056
			(−3.064)	(−0.074)
**Constant**	5.987***	4.735***	7.168***	5.255***
	(123.005)	(177.237)	(12.636)	(16.947)
**Individual-fixed effects**	YES	YES	YES	YES
**Year-fixed effects**	YES	YES	YES	YES
**Observations**	210	210	210	210
**R-squared**	0.909	0.966	0.919	0.970

Note

***, **, *indicate significance at 1%, 5%, and 10%, respectively. The same applies to the following tables.

Based on the results in columns (3) and (4) of Table 3, we analyze the magnitude of the effects of the digital economy on the social insurance funds revenue. In columns (3) and (4), the estimated values of the *Dige* are 0.555 and 0.332, respectively, and both are significantly positive at the 1% statistical level. These results indicate that for every one unit increase in the development of the digital economy, the revenue of the basic endowment insurance funds increases by 0.56% and the revenue of the basic health insurance funds increases by 0.33%. These results suggest that the digital economy has the potential to increase the social insurance funds revenue. In an aging society, increasing the revenue of social insurance funds is crucial to meet the growing expenditure needs and ensure the sustainability of the social insurance system.

The coefficients of the variable *Ind* in columns (3) and (4) are significantly negative, indicating that the more advanced the industrial structure, the lower the revenue of the endowment and health insurance funds, which is inconsistent with our theoretical analysis. The possible reason for this is that although the industrial structure tends to more advanced as the proportion of China’s tertiary industry increases, the quality of China’s tertiary industry development is low. Most enterprises in the service sector are small and medium-sized and self-employed, and due to the lack of effective supervision, the participation rate of these enterprises is even lower than that of the secondary industry. Wang et al. [34] also found a negative relationship between industrial structure and social insurance participation. The coefficient on the variable *Ar* is significantly negative in column (3) but no longer significant in column (4), suggesting that the estimated coefficient on *Ar* lacks robustness. The estimated coefficient of the variable *Up* in Table 3 is negative, but insignificant, which means that the estimated coefficient of the variable cannot reject the hypothesis of zero. A possible reason for this is the existence of the household registration barrier in China, whereby urban residents with rural household registration cannot enjoy social security benefits in urban areas.

### 4.3 Robustness test

#### 4.3.1 Determining the weights of the digital economy indicators using principal component analysis method

Variable measurement error can lead to estimation bias. The measurement method of variables can be one of the sources of variable measurement error. In the benchmark regression, this paper adopts the entropy value method to assign weights to the digital economy indicators, but the entropy value method is only one of the weighting methods. In order to avoid the variable measurement error caused by the selection of the weighting method, this paper uses the principal component analysis method to reweight the digital economy indicators. Based on the new value of the digital economy development index, the impact of the digital economy on the revenue of the endowment insurance funds and the health insurance funds is estimated, and the results are shown in columns (1) and (2) of Table 4. The regression coefficients of *Dige* in columns (1) and (2) are significant at the 5% and 1% statistical levels, with values of 0.097 and 0.064, respectively. These results indicate that the benchmark regression results are robust and not affected by measurement errors of the variables.

**Table 4:** Robustness test.

Variables	(1)	(2)	(3)	(4)	(5)
	Principal component analysis method		Instrumental variable method		
			First stage	Second stage	
	*EIFR*	*HIFR*	*Dige*	*EIFR*	*HIFR*
***Dige***	0.097**	0.064***		2.127***	1.640***
	(2.515)	(3.057)		(5.706)	(7.009)
***Ind***	−0.194**	−0.186***		−0.200	−0.225**
	(−2.486)	(−4.373)		(−1.323)	(−2.291)
***Doow***	0.054	0.042		−0.465**	−0.184
	(0.567)	(0.818)		(−2.272)	(−1.446)
***Fin***	−0.509	−0.237		−1.194	−0.101
	(−0.817)	(−0.698)		(−1.027)	(−0.145)
***Up***	−1.379	−0.776*		−3.842*	−2.014
	(−1.619)	(−1.673)		(−1.798)	(−1.512)
***Ar***	−4.296***	−0.106		−10.786***	−4.462**
	(−3.050)	(−0.139)		(−3.081)	(−2.005)
**IV**			0.525***		
			(6.526)		
**Constant**	7.587***	5.513***			
	(13.492)	(18.010)			
**Individual-fixed effects**	YES	YES	YES	YES	YES
**Year-fixed effects**	YES	YES	YES	YES	YES
**Kleibergen-Paap rk LM Statistics**			16.70		
			[0.000]		
**Kleibergen-Paap rk Wald F Statistics**			31.63		
			[16.38]		
**Hansen J Statistics**			0.000		
**Observations**	210	210	203	203	203
**R-squared**	0.919	0.970		0.722	0.866

#### 4.3.2 Instrumental variable method

Although we control as much as possible for the factors that affect the revenue of the social insurance funds, it is still possible that the omission of important variables could lead to endogeneity problems, resulting in biased results in the benchmark estimation. This paper used the instrumental variable method to address endogeneity problems that may arise from omitted variables. Following Pang et al. [38] and Kong and Li [39], the number of fixed-line telephones per 100 residents in each province in 1984 was used as the instrumental variable for the digital economy. Due to the cross-sectional nature of this instrumental variable, it cannot be used directly in panel data econometric analysis. Therefore, the time-varying variables were introduced to construct the panel instrumental variable following the method of Nuun and Qian [40]. Specifically, the number of Internet users in the last year and the number of fixed-line telephones per 100 residents in 1984 were used to generate the interaction term, which was then logged to serve as the instrumental variable for the digital economy development.

The results of the instrumental variable method regression based on two-stage least squares (2SLS) are reported in columns (3)-(5) of Table 4. The Kleibergen-Paap rk LM statistic has a p-value of 0.000, indicating the initial hypothesis of non-identifiability is rejected. Since the Kleibergen-Paap RK Wald F statistic is higher than the threshold value of 16.38, the model does not have the problem of weak instrumental variables. As the P-value of the Hansen J statistic is 0, the result cannot reject the null hypothesis that "all instrumental variables are exogenous."

The regression coefficients of *Dige* are all positive and significant at the 1% level according to the results of the second stage regression. An increase of one unit in the development of the digital economy increased the revenue of basic endowment insurance funds by 2.22% and the revenue of basic health insurance funds by 1.64%. The effect of digital economy on revenue growth persisted even after accounting for possible endogeneity.

### 4.4 Mechanisms

#### 4.4.1 Employment mechanism

Given the contribution rate, the participation rate and contributions per capita determine the revenue of the social insurance funds. As the digital economy grows, so does the employment rate, which increases the participation rate and contributions per capita, and thus the revenue of the social insurance funds. The results of the mechanism test on employment are shown in Table 5.

**Table 5:** Mechanism test: Employment.

Variables	(1)	(2)	(3)	(5)	(6)
	*Emp*	*PIPR*	*HIPR*	*PICR*	*HICR*
***Dige***	0.073*				
	(1.936)				
***Emp***		0.139**	0.018	0.348**	0.128
		(2.013)	(0.330)	(2.080)	(1.173)
***Ind***	−0.009	−0.028**	−0.023**	−0.000	0.011
	(−0.630)	(−2.108)	(−2.120)	(−0.002)	(0.547)
***Doow***	−0.030*	0.009	−0.000	−0.065	−0.050*
	(−1.657)	(0.537)	(−0.015)	(−1.624)	(−1.925)
***Fin***	−0.048	−0.034	−0.145*	−0.017	−0.108
	(−0.391)	(−0.321)	(−1.667)	(−0.066)	(−0.640)
***Up***	−0.479***	−0.691***	−0.584***	−0.985***	−0.784***
	(−2.901)	(−4.522)	(−4.715)	(−2.661)	(−3.259)
***Ar***	0.105	−0.051	0.512***	0.524	0.526
	(0.387)	(−0.209)	(2.606)	(0.892)	(1.378)
**Constant**	0.936***	0.519***	0.499***	0.857***	0.524***
	(8.516)	(4.367)	(5.180)	(2.980)	(2.803)
**Individual-fixed effects**	YES	YES	YES	YES	YES
**Year-fixed effects**	YES	YES	YES	YES	YES
**Observations**	210	210	210	210	210
**R-squared**	0.262	0.724	0.475	0.834	0.819

According to column (1), the employment rate is positively affected by the digital economy with a coefficient of 0.073, which is statistically significant at the 10% level. Columns (2)-(5) present the results of the effects of the employment rate on various variables, including the basic endowment insurance participation rate (*PIPR*), basic health insurance participation rate (*HIPR*), basic endowment insurance contributions per capita(*PICR*), and basic health insurance contributions per capita (*HICR*). The employment rate significantly increased the basic endowment insurance participation rate and basic endowment insurance contributions per capita. However, it had no significant effect on the basic health insurance participation rate and basic health insurance contributions per capita. These results suggest that the employment mechanism was only valid for the basic endowment insurance and not for the basic health insurance.

#### 4.4.2 Income mechanism

The willingness to participate in social insurance and the amount of contributions are significantly influenced by income [41]. The digital economy increases the social insurance participation rate and contributions per capita by increasing income, thus increasing the social insurance funds revenue.

Table 6 shows the results of the income mechanism. As can be seen in column (1), the digital economy and the average wage are positively correlated and significant at the 1% statistical level, indicating that the digital economy contributes to increasing income. The results in columns (2)-(4) show that the average wage contributes significantly to the participation rate and the contributions per capita to the basic endowment insurance and the basic health insurance. Based on the results in Table 6, the income mechanism holds for both the basic endowment insurance and the basic health insurance.

**Table 6:** Mechanism test: Income.

Variables	(1)	(2)	(3)	(4)	(5)
	*Aw*	*PIPR*	*HIPR*	*PICR*	*HICR*
***Dige***	0.276***				
	(3.276)				
***Aw***		0.083***	0.073***	0.317***	0.249***
		(2.744)	(3.014)	(4.486)	(5.623)
***Ind***	0.034	−0.032**	−0.026**	−0.017	−0.001
	(1.056)	(−2.476)	(−2.498)	(−0.551)	(−0.053)
***Doow***	−0.055	0.009	0.002	−0.062	−0.044*
	(−1.342)	(0.524)	(0.174)	(−1.607)	(−1.815)
***Fin***	−0.255	−0.010	−0.113	0.094	−0.005
	(−0.946)	(−0.096)	(−1.326)	(0.378)	(−0.035)
***Up***	0.373	−0.782***	−0.610***	−1.239***	−0.908***
	(1.016)	(−5.311)	(−5.196)	(−3.591)	(−4.208)
***Ar***	−1.239**	0.053	0.584***	0.886	0.783**
	(−2.060)	(0.218)	(3.032)	(1.568)	(2.215)
**Constant**	9.918***	−0.179	−0.214	−1.994***	−1.854***
	(40.615)	(−0.562)	(−0.840)	(−2.671)	(−3.969)
**Individual-fixed effects**	YES	YES	YES	YES	YES
**Year-fixed effects**	YES	YES	YES	YES	YES
**Observations**	210	210	210	210	210
**R-squared**	0.964	0.730	0.502	0.848	0.847

### 4.5 Heterogeneity analysis

#### 4.5.1 Heterogeneity in economic development

For regions with different levels of economic development, there may be differences in the positive effects of the digital economy on the social insurance funds revenue. Digital technology provides development opportunities for economically underdeveloped regions by breaking down technical and market barriers to the circulation of factors between regions [42], so that economically underdeveloped regions realize a greater increase in social insurance funds revenue by taking advantage of the latecomer advantage.

GDP per capita was used as a measure of economic development in this paper. The sample was divided into regions of higher and lower economic development according to the median GDP per capita of the sample in each year. Sub-sample regressions were run and the results are shown in Table 7. The results in columns (1) and (2) show that, in regions with higher economic development, the higher the development of the digital economy, the higher the revenue of the basic endowment insurance funds. The results in columns (3) and (4) show that in regions with lower economic development, the development of the digital economy has a significant positive effect on the revenue of both the basic endowment insurance funds and the basic health insurance funds. For the results in columns (1) and (3), the coefficient of the digital economy in column (3) is larger than in column (1), which proves that the contribution of the development of the digital economy to social insurance funds revenue is more remarkable in regions with lower economic development than in regions with higher economic development.

**Table 7:** Heterogeneity analysis: The economic development.

Variables	(1)	(2)	(3)	(4)
	High-level		Low-level	
	*EIFR*	*HIFR*	*EIFR*	*HIFR*
***Dige***	0.573***	0.191	1.019**	0.578***
	(2.807)	(1.355)	(2.269)	(2.661)
***Ind***	−0.059	−0.053	−0.403***	−0.216***
	(−0.464)	(−0.600)	(−2.952)	(−3.275)
***Doow***	−0.027	0.022	0.623	0.057
	(−0.299)	(0.360)	(1.217)	(0.229)
***Fin***	0.602	−0.603	0.121	0.300
	(0.712)	(−1.030)	(0.125)	(0.643)
***Up***	0.176	−1.137	−6.807***	0.344
	(0.152)	(−1.424)	(−3.086)	(0.323)
***Ar***	−0.038	−0.778	−10.175***	0.543
	(−0.025)	(−0.749)	(−4.059)	(0.448)
**Individual-fixed effects**	YES	YES	YES	YES
**Year-fixed effects**	YES	YES	YES	YES
**Constant**	6.028***	6.026***	9.776***	4.275***
	(6.814)	(9.843)	(9.280)	(8.392)
**Observations**	105	105	105	105
**R-squared**	0.941	0.969	0.933	0.977

#### 4.5.2 Heterogeneity of urbanization

The higher the urbanization, the wider the social insurance coverage. The digital economy is conducive to promoting rural-urban migration, and the urbanization rate may be faster in areas with low urbanization. Therefore, the digital economy may play a more vital role in promoting social insurance funds revenue in areas with low urbanization. The sample was divided into high and low urbanization samples according to the median urbanization in each year. The results are shown in Table 8. Columns (1) and (2) are the regression results of the high-urbanization sample, and columns (3) and (4) are the regression results of the low-urbanization sample. The digital economy significantly affected the revenue of the basic endowment insurance funds and the basic health insurance funds. However, the digital economy had a more significant effect on promoting social insurance funds revenue in low-urbanization areas than in high-urbanization areas.

**Table 8:** Heterogeneity analysis: Urbanization.

Variables	(1)	(2)	(3)	(4)
	High-urbanization		Low-urbanization	
	*EIFR*	*HIFR*	*EIFR*	*HIFR*
***Dige***	0.542***	0.235*	0.823*	0.656***
	(2.686)	(1.719)	(1.720)	(2.718)
***Ind***	−0.143*	−0.114**	−0.356**	−0.246***
	(−1.809)	(−2.124)	(−2.224)	(−3.055)
***Doow***	−0.015	0.045	−0.205	0.329
	(−0.162)	(0.727)	(−0.359)	(1.144)
***Fin***	0.019	−0.633	−0.088	0.420
	(0.022)	(−1.072)	(−0.082)	(0.775)
***Up***	−0.961	−1.744**	−4.760*	0.556
	(−0.942)	(−2.519)	(−1.827)	(0.423)
***Ar***	−0.564	−0.857	−10.501***	0.397
	(−0.387)	(−0.865)	(−3.734)	(0.280)
**Individual-fixed effects**	YES	YES	YES	YES
**Year-fixed effects**	YES	YES	YES	YES
**Constant**	6.991***	6.476***	8.962***	4.141***
	(8.935)	(12.198)	(7.637)	(7.002)
**Observations**	105	105	105	105
**R-squared**	0.939	0.970	0.927	0.974

## 5. Discussion

Several countries, including China, are facing a crisis in the balance of social insurance funds [37, 43]. The widespread use of digital technologies has significantly influenced economic and social development [44, 45]. Understanding the impact of the digital economy on social insurance funds has become an important issue in the digital era. China, in particular, is experiencing rapid aging and faces the challenge of balancing its social insurance funds. At the same time, China’s digital economy has made remarkable progress in recent years, both in terms of speed and scale. These unique characteristics make it an ideal case to study the relationship between the digital economy and social insurance funds revenue. In our study, we conducted a comprehensive review of the existing literature, constructed a two-way fixed effects model, and empirically examined the effects of the digital economy on social insurance funds revenue using data from 30 provinces in China from 2011 to 2017.

Our benchmark regression results indicated that the development of the digital economy had a positive impact on social insurance funds revenue. An increase of one unit in the digital economy development index contributed to an increase of 0.56% and 0.33% in endowment and health insurance funds respectively. To ensure the credibility of our findings, we conducted robustness tests by changing the weighting method and using instrumental variables. These tests confirmed the credibility of our study. By addressing concerns raised in the existing literature, we shed light on how the digital economy can increase social insurance funds revenue and provide valuable policy recommendations.

The mechanism analysis revealed that the digital economy facilitated employment and increased workers’ wages, which is consistent with the previous studies [46, 47]. However, concerns have been raised about technological unemployment and flexible employment caused by the digital economy, which may reduce the number of social insurance contributors and threaten the sustainability of social insurance funds. Our findings indicated that the digital economy, despite causing technological unemployment and flexible employment [11], the digital economy has actually led to a net increase in formal employment [14] and increased social insurance funds revenue.

## 6. Conclusion

The effects of the digital economy on social insurance funds revenue not only contribute to our understanding of the role of the digital economy, but are also crucial for ensuring the sustainable development of social insurance in an aging society. Using data from 30 provinces in China between 2011 and 2017, this study examined the impact of the digital economy on the revenue of basic endowment insurance funds and basic health insurance funds on a two-way fixed effects model.

The results showed that the development of the digital economy led to an increase in social insurance funds revenue. For every one percent increase in the development of the digital economy, the revenue of the basic endowment insurance funds increased by 0.56%, and the revenue of the basic health insurance funds increased by 0.33%. The mechanism analysis revealed that employment and income were the two main channels through which the digital economy affected the social insurance funds revenue. The heterogeneity analysis indicated that the positive effects of the digital economy on social insurance funds revenue were stronger in regions with lower levels of economic development and urbanization.

Several policy implications can be drawn from these findings. The government can stimulate the development potential of the digital economy to increase the social insurance funds revenue, especially in less economically developed cities or areas with lower urbanization rates. The construction of digital infrastructure should be prioritized, with increased allocation of fiscal resources, especially less in economically developed regions. Additionally, the spillover effects of the digital economy can be maximized through enhanced inter-regional and inter-industry technology exchange and cooperation [39].

To mitigate the potential negative impact on social insurance funds revenue, we should pay attention to the participation issue of the unemployed and those engaged in flexible employment due to digital transformation. Efforts should be made to help the technically unemployed reintegrate into the workforce through digital skills training. Furthermore, incentive mechanisms can be put in place to encourage flexible workers to actively contribute.

However, there are several limitations to this study. First, there is no unified standard for measuring the digital economy, and although we used a relatively high quality indicator system, further validation is needed. Second, due to the lack of data on the number of employees, wages, and social insurance contributions of flexible employment, we were unable to examine the applicability of employment and income mechanisms to flexible employment or to analyze the contribution behaviors of individuals engaged in flexible employment. It is worth noting that flexible employment serves as a "reservoir", where some individuals eventually transition return to formal employment after a short adjustment period. In addition, in China, individuals engaged in flexible employment can still contribute to basic endowment insurance and basic health insurance premiums.

In terms of future research directions, it would be valuable to study the behavioral mechanisms through which the digital economy affects social insurance funds revenue from the perspective of behavioral economics. Additionally, it is crucial to study the impact of the digital economy on the social insurance funds expenditure. The digital economy not only affects revenue but may also affect funds expenditures [48]. A comprehensive analysis of both revenue and expenditure can provide insights into the sustainability of social insurance funds in the context of the digital economy.

## Supporting information

S1 Data (XLSX)
